# Physiological efficacy of the amino acid-based biostimulants Pepton 85/16, Pepton origin, and Nutriterra in lettuce grown under optimal and reduced synthetic nitrogen fertilization

**DOI:** 10.3389/fpls.2025.1645768

**Published:** 2025-09-08

**Authors:** Santiago Atero-Calvo, Eloy Navarro-León, Javier Polo, Juan Manuel Ruiz

**Affiliations:** ^1^ Department of Plant Physiology, Faculty of Sciences, University of Granada, Granada, Spain; ^2^ APC Europe, Granollers, Spain

**Keywords:** amino acids, biostimulants, humic acids, nitrate, nitrogen metabolism, photosynthesis, protein hydrolysates

## Abstract

Improving nitrogen (N) use efficiency (NUE) is a key objective in sustainable agriculture, particularly for leafy vegetables such as lettuce, which require high N inputs. Biostimulants offer a promising strategy for enhancing crop performance while reducing fertilizer dependency. In this study, we evaluated the effects of three amino acid-based biostimulants, Pepton 85/16, Pepton Origin, and Nutriterra, on lettuce growth and NUE under three N fertilization levels (100%, 70%, and 40% of the recommended dose). All biostimulants improved shoot biomass, leaf area, and physiological performance, including photosynthetic rate (*A*) and key N assimilation parameters. Nutriterra was the most effective under full N supply, enhancing both productivity and water use efficiency (WUE) while reducing leaf nitrate concentration, contributing to improved crop quality. Under N-limited conditions, Pepton 85/16 consistently outperformed the other products, especially at 70% N, where it restored and even exceeded the biomass levels observed under optimal N. This effect was associated with enhanced nitrate reductase (NR) and glutamine synthetase (GS) activity, increased protein and amino acid concentrations, and higher organic N levels. While Pepton Origin also showed beneficial effects under N limitation, its impact was less pronounced. Overall, the targeted use of these biostimulants represents an effective strategy for maintaining productivity and reducing synthetic N fertilization in lettuce cultivation systems.

## 1 Introduction

Nitrogen (N) is one of the major essential nutrients in plants, typically representing between 1.5% and 6% of dry matter (Marschner, 2011). It is present in three main forms: over 50% is incorporated into high-molecular-weight compounds such as proteins and nucleic acids, while the remainder exists as soluble organic N (including amino acids, amides, and amines) or as inorganic N, primarily nitrate and ammonium ions (Lea and Azevedo, 2006). N is also crucial in the biochemistry of non-protein compounds such as coenzymes, photosynthetic pigments, secondary metabolites, and polyamines (Stitt and Krapp, 1999). These physiological and nutritional characteristics make N a critical element for achieving optimal yields in most crops (de Bang et al., 2021).

Currently, the significance of N in agriculture is heightened because of the dominant focus on maximizing production. Rapid demographic growth in recent decades has increased the need for higher crop yields for human consumption. This demand must be met without expanding the cultivated area, as agriculture already occupies most of the fertile land and uses a large share of resources such as water and fertilizers (Lawlor, 2002). As a result, synthetic N fertilizers have become a cornerstone of intensive farming systems, with global consumption surpassing 100 million tons in 2018 (Dimkpa et al., 2020; Javed et al., 2022; Li et al., 2022).

However, the efficiency of N fertilizers is far from optimal. The nitrogen use efficiency (NUE) in agricultural systems generally ranges between 40% and 70% (Zhang et al., 2015), meaning that a considerable fraction of applied N is not taken up by crops. This unrecovered N is lost through volatilization, leaching, and nitrification, leading to decreased productivity and severe environmental consequences (Heffer and Prud’homme, 2015; Li et al., 2022). Inefficient fertilizer use contributes to nitrate leaching into water bodies, eutrophication, greenhouse gas emissions, and the accumulation of nitrate in edible plant tissues, which poses health risks due to its potential conversion into nitrite and carcinogenic N-nitroso compounds (Mensinga et al., 2003).

Beyond these impacts, excessive fertilizer application is also a major driver of soil degradation, particularly salinization. Continuous and unbalanced use of synthetic fertilizers increases soil electrical conductivity, alters ionic balance, and accelerates the accumulation of soluble salts in the rhizosphere (Qadir et al., 2014). Soil salinization currently affects more than 20% of irrigated lands worldwide and represents a serious threat to sustainable food production (Shrivastava and Kumar, 2015; Gatti et al., 2025). This highlights the need to develop agronomic strategies that improve NUE while minimizing the negative effects of fertilizer overuse, including soil salinization and broader environmental pollution.

Strategies to enhance NUE include optimizing fertilizer dose, timing, and chemical form, as well as adopting agronomic techniques (Zhang et al., 2015). In this context, biostimulants represent a promising strategy to enhance NUE and support sustainable nutrient management. NUE is defined as biomass produced per unit of available N, and it is composed of two key components: N uptake efficiency (NUpE), which refers to the plant’s ability to absorb N from the soil, and N utilization efficiency (NUtE), which describes how effectively the absorbed N is used to produce biomass in various plant organs (Xu et al., 2012; Dimkpa et al., 2020; Javed et al., 2022). Although genetic and biotechnological approaches have been explored to improve NUE, these solutions are often time-consuming and resource-intensive. For this reason, there is growing interest in fast-acting, environmentally friendly, and easy-to-apply alternative strategies, such as the use of biostimulants (Ciampitti et al., 2022; Ali et al., 2025).

Over the past 25 years, biostimulants derived from natural raw materials have received increasing attention from researchers and agricultural industries. These substances have a novel approach that influences plant physiological processes to stimulate growth, alleviate stress-induced limitations, and improve yield (Brown and Saa, 2015; du Jardin, 2015; Zulfiqar et al., 2020). Among the biostimulants available on the market, one type consists of amino acid-based products obtained through chemical or enzymatic hydrolysis of plant or animal proteins (Sun et al., 2024). These biostimulants have shown beneficial effects on plant growth by contributing to the biosynthesis of a wide range of N-containing non-protein compounds, such as pigments, vitamins, coenzymes, purines, and pyrimidines (Francesca et al., 2020; Sun et al., 2024; Gatti et al., 2025).

The potential functions of amino acid-based biostimulants in plants include the following: improving nutrient uptake by acting as natural chelating agents that increase nutrient bioavailability in the soil; modifying root architecture by promoting higher density, length, and number of lateral roots; enhancing nutrient use efficiency, especially N, by stimulating nitrate assimilation and thereby reducing leaf nitrate accumulation; exerting or inducing hormonal activity in plants; and increasing resistance to environmental stress by promoting the synthesis of antioxidant and osmoprotective compounds (Francesca et al., 2020; Ciriello et al., 2024; Sun et al., 2024).

The influence of these biostimulants on N metabolism and NUE is another significant aspect. Although limited, some research has demonstrated their potential to improve growth and productivity by enhancing N assimilation. For example, studies on spinach and lettuce have shown that these biostimulants can stimulate enzymes such as nitrate reductase (NR) and glutamine synthetase (GS), which are key regulators of N assimilation (Ottaiano et al., 2021). Furthermore, investigations into the application method have revealed that root application is often more effective than foliar treatment. Specifically, root-applied amino acid biostimulants more strongly induce plant growth, leaf area development, and N assimilation processes, as observed in lettuce and tomato plants (Cristofano et al., 2021; Choi et al., 2022).

Therefore, the objective of the present study is to evaluate the effects of root-applied amino acid-based biostimulants, developed by APC Europe SLU, on growth, N assimilation, and NUE in lettuce plants grown under different N fertilization levels (100%, 70%, and 40% N of the recommended dose). Specifically, we aimed to determine their influence on plant growth, photosynthetic performance, N assimilation, and NUE indices. Our hypothesis was that the application of these biostimulants could enhance NUE and photosynthetic efficiency, particularly under conditions of reduced synthetic N fertilization, thereby providing a sustainable approach to maintaining crop productivity while reducing chemical fertilizer inputs.

## 2 Materials and methods

### 2.1 Plant material and growth conditions

Lettuce plants (*Lactuca sativa* cv. Isasa) were used in this study. Seeds were germinated in a commercial nursery (Saliplant S.L., Carchuna, Granada, Spain) in seedling trays with individual cells (3 cm × 3 cm × 10 cm) and grown for 45 days under standard nursery conditions. After this period, each seedling was transferred to an individual plastic pot (13 cm top diameter, 10 cm bottom diameter, 12.5 cm height, and a total volume of 2 L) filled with a 1:1 mixture of vermiculite and perlite and placed in a growth chamber in the Department of Plant Physiology at the University of Granada. The plants were grouped in plastic trays (57 cm × 41 cm × 7 cm). In the grow chamber, plants grew under controlled environmental conditions: relative humidity between 60% and 80%, temperature of 25°C during the day and 15°C at night, and a 16-hour light/8-hour dark photoperiod. The photosynthetic photon flux density (PPFD) was 350 µmol m^−^² s^−^¹, measured with an SB Quantum 190 sensor (LI-COR Inc., Lincoln, NE, USA).

Plants were irrigated as described by Atero-Calvo et al. (2024a) with a modified Hoagland nutrient solution specifically adapted for lettuce cultivation, ensuring adequate macronutrient and micronutrient supply. The solution contained: 10 mM NaNO_3_, 4 mM KCl, 4 mM CaCl_2_, 2 mM MgSO_4_, 2 mM NaH_2_PO_4_, 2 µM MnCl_2_, 1 µM ZnSO_4_, 0.25 µM CuSO_4_, 0.1 µM Na_2_MoO_4_, 125 µM Fe-EDDHA, and 50 µM H_3_BO_3_, adjusted to a pH of 5.8. The solution was renewed every 3 days, and 1.5 L was applied to the trays each time.

### 2.2 Treatment description and experimental design

This experiment evaluated the effect of three biostimulant products (Pepton 85/16, Pepton Origin, and Nutriterra) supplied by APC Europe SLU applied in combination with different N fertilization levels.

The three N levels were achieved by adding NaNO_3_ (Panreac Química S.L.U., Barcelona, Spain) to the nutritive solution at different doses: 10 mM N (N - 100%), 7 mM N (N - 70%), and 4 mM N (N - 40%). The N doses were selected following previous studies of our research group in lettuce (Navarro-León et al., 2022). These treatments were applied through the nutrient solution. Thus, the plants of the N - 100% treatments received the nutrient solution described above, and the plants of the N - 70% and N - 40% treatments received the same nutrient solution but with the corresponding reductions in NaNO_3_. For each N dose, a control treatment without the application of biostimulants was performed. The treatments used are summarized in **Table 1**.

**Table 1:** Description and total N content of the treatments used in the study.

Treatments	Description	Total N content (mg/L)
T1	N-100%	140
T2	N-100% + Biostimulant_1 APC Agro PEPTON 85/16 equivalent dose 2 g/L	166
T3	N-100% + Biostimulant_2 APC Agro PEPTON ORIGIN equivalent dose 2 g/L	168
T4	N-100% + Biostimulant_3 APC Agro NUTRITERRA equivalent dose 6 mL/L	144.6
T5	N-70%	98
T6	N-70% + Biostimulant_1 APC Agro PEPTON 85/16 equivalent dose 2 g/L	124
T7	N-70% + Biostimulant_2 APC Agro PEPTON ORIGIN equivalent dose 2 g/L	126
T8	N-70% + Biostimulant_3 APC Agro NUTRITERRA equivalent dose 6 mL/L	102.6
T9	N-40%	56
T10	N-40% + Biostimulant_1 APC Agro PEPTON 85/16 equivalent dose 2 g/L	82
T11	N-40% + Biostimulant_2 APC Agro PEPTON ORIGIN equivalent dose 2 g/L	84
T12	N-40% + Biostimulant_3 APC Agro NUTRITERRA equivalent dose 6 mL/L	60.4

The biostimulants composition is detailed in **Supplementary Table S1**. The biostimulant Pepton 85/16 is a micro-granulated characterized by its high total amino acid content (85%), free amino acids (16%), potassium oxide content (5%), and the presence of magnesium oxide (0.04%). Pepton origin is a micro-granulated biostimulant that contains the highest total amino acid content (93%) and free amino acids (40%) and the highest levels of calcium oxide (0.04%) and iron (0.27%). Finally, Nutriterra is a liquid biostimulant with lower total amino acid content (13.5%) but contains 14% humic substances and the highest phosphorus pentoxide content (3%).

The application of treatments began 45 days after seed germination (18 February 2025), and the biostimulants were applied four times at weekly intervals, following the recommendations of the R&D team of APC Europe SLU’s. The biostimulants were applied together with the nutritive solution at a dose of 2 g/L for Pepton 85/16 and Pepton origin, and 6 mL/L for Nutriterra.

The experimental design was a complete randomized block design. For each treatment, a total of 18 plants (n = 18) were arranged in three trays containing six plants each. Each plant was grown in an individual pot, and trays were randomly distributed within the growth chamber and repositioned every 3 days to minimize positional effects.

### 2.3 Plant sampling

Plant sampling was performed 3 days after the fourth biostimulant application (March 14, 2025). All plants from each treatment group were immediately processed for subsequent analysis. The plant material was surface-cleaned, blotted dry on filter paper, and fresh weight (FW) was recorded. Half of the fresh material, either immediately processed or frozen at –40°C, was used for the analysis of the following parameters: leaf area, photosynthetic efficiency measured with an Infra-Red Gas Analyzer, N metabolism enzyme activities (NR and GS), and amino acids and soluble proteins concentrations. The remaining plant material was oven-dried using forced air circulation and used to determine dry weight (DW), nitrate and ammonium concentrations, total N content, and different NUE parameters.

### 2.4 Plant material analysis

#### 2.4.1 Leaf area

The leaf area was measured using a LI-COR optical reader, model LI - 3000A (LI-COR Inc., Lincoln, NE, USA).

#### 2.4.2 Gas exchange parameters

Measurements were recorded using a LI-COR 6800 portable photosynthesis system infrared gas analyzer (LI-COR Inc., Lincoln, NE, USA) as described by Atero-Calvo et al. (2024b). The intermediate leaves were placed in the measuring chamber under optimal growth conditions. Before use, the instrument was warmed up for 30 minutes and calibrated. Standard optimal chamber conditions were set at 500 μmol m^−^² s^−^¹ of photosynthetically active radiation (PAR), 400 μmol mol^−^¹ CO_2_ concentration, 30°C leaf temperature, and 60% relative humidity. The net photosynthetic rate (*A*), transpiration rate (*E*), stomatal resistance (r), and intracellular concentration of CO_2_ (Ci) were simultaneously recorded. Data were stored on the LI-COR device and analyzed using “Photosyn Assistant” software. The instantaneous water use efficiency (WUE) was calculated by dividing *A* by the corresponding *E*.

#### 2.4.3 Enzymatic activities of N metabolism

##### 2.4.3.1 Nitrate reductase activity

The NR activity was determined following the procedure described by Navarro-León et al. (2016). Fresh leaf tissue (0.2 g) was ground in a mortar with 1 mL of extraction buffer containing 2 mM EDTA-Na, 2 mM DTT, 1% (w/v) PVPP in 100 mM KH_2_PO_4_ (pH 7.5). The homogenate was centrifuged for 20 min at 20,600 g at 4°C. The supernatant was added to a reaction mixture containing 100 mM KNO_3_, 2 mM NADH, 10 mM cysteine, and 10 mM MgCl_2_ in 100 mM KH_2_PO_4_ buffer (pH 7.5) and incubated at 30 °C for 30 min. Zinc acetate (1 mM) was added as a stopping reagent, and nitrite (NO_2_^−^) formation was detected with 1% sulfanilamide in 1.5 M HCl and 0.02% (w/v) NNEDA in 0.2 M HCl. The absorbance at 540 nm was used to determine NR activity.

##### 2.4.3.2 Glutamine synthetase activity

Glutamine synthetase (GS) activity was determined using an adaptation of the hydroxamate synthetase assay described by Navarro-León et al. (2016). Leaf tissue (0.1 g) was ground in a mortar with 1 mL of extraction buffer containing 100 mM sucrose, 2% (v/v) β-mercaptoethanol, and 20% (v/v) ethylene glycol in 100 mM malic acid-KOH (pH 6.8). The homogenate was centrifuged at 20,600 g for 20 min at 4°C. The resulting extract was used to determine GS activity. The reaction mixture contained 150 mM sodium glutamate, 30 mM hydroxylamine, and 10 mM ATP as substrates, along with 45 mM MgSO_4_•7 H_2_O and 4 mM EDTA-Na in 150 mM imidazole-HCl buffer (pH 7.8). After incubation at 28°C for 30 min, the formation of γ-glutamyl hydroxamate was determined by measuring the absorbance at 540 nm after its reaction with acidified ferric chloride.

#### 2.4.4 Determination of soluble amino acids and proteins

To determine soluble amino acids and proteins, approximately 0.5 g of plant tissue was homogenized in 5 mL of 50 mM phosphate buffer (pH 7.0). The homogenate was filtered through four layers of gauze and centrifuged at 12,360 g for 15 minutes. The supernatant was used for quantification. The soluble amino acids were quantified using the ninhydrin method. For soluble proteins, 0.1 mL of the supernatant was mixed with 0.9 mL of 50 mM phosphate buffer (pH 7.0) and 5 mL of Coomassie Brilliant Blue reagent. After 20 minutes, the absorbance was measured at 595 nm and compared with a standard curve prepared with bovine serum albumin (Navarro-León et al., 2016).

#### 2.4.5 Nitrate, ammonium and total N concentration

Soluble NO_3_^−^ and NH_4_^+^ concentrations were determined using aqueous extraction according to the method of Cataldo et al. (1975). NO_3_^−^ determination was based on the colorimetric reaction between nitrate and salicylic acid under alkaline conditions. NH_4_^+^ concentration was determined using the Berthelot reaction following the method described by Krom (1980). The total N concentration was determined via elemental analysis (Leco Truspec CN) based on the complete and instantaneous oxidation of the dry sample.

#### 2.4.6 N use efficiency parameters

The NUE parameters were calculated using the formulas defined by Dobermann (2007), which form the basis of the European Biostimulants Regulation FprCEN/TS 17700 - 2. These parameters integrate plant growth, N uptake, and N utilization to evaluate the efficiency of applied nitrogen. Calculations were based on the biomass of the aerial part (shoot DW), nitrogen concentration in the corresponding tissues, and the amount of N applied through fertilization. The parameters were determined as follows:

- Apparent N recovery efficiency (RE). Indicates the proportion of applied N recovered by the plant. It is expressed as the increase in N uptake (mg) per mg of N applied

$$RE=\;\frac{\left[\text{N}\right]\text{ Treated plant }-\;\left[\text{N}\right]\text{ Control plant}}{\left[\text{N}\right]\text{ supplied through fertilization}+\text{biostimulants }}$$

where [N] is the N content in mg.

- Nutrient export (NE). Represents the amount of N accumulated in the shoot or a specific organ. It is expressed as mg of N.

$$NE=\text{Biomass }\left(\text{g DW}\right)\times {\text{[N concentration in organ or whole plant (mg g}}^{-1}\text{)]}$$

- Internal N utilization efficiency (IE). Reflects the plant’s ability to convert absorbed N into biomass. It is expressed as g² DW mg^−1^ N.

$$IE=\;\frac{\text{Biomass }\left(\text{g DW}\right)}{\left[\text{N content in shoot }\left(\text{mg}\right)\right]\;}$$

- Agronomic efficiency of applied N (AE). Measures the increase in biomass that produces a treatment in comparison to a control relative to the nitrogen applied. It is expressed as g DW mg^−1^ N applied.

$$AE=\;\frac{Biomass_{treatment}\;-\;Biomass_{control}}{\left[\text{N}\right]\text{supplied through fertilization}+\text{biostimulants }}$$

- Partial Factor Productivity of Applied N (PFP). Indicates overall productivity per unit of applied N. It is expressed as g DW mg^−1^ N applied.

$$PFP=\;\frac{\text{Biomass }\left(\text{g DW}\right)}{\left[\text{N}\right]\text{supplied through fertilization}+\text{biostimulants }}$$

### 2.5 Statistical analysis

All analyses were performed in triplicate, and the results were statistically evaluated using one-way analysis of variance (ANOVA) with a 95% confidence interval. Differences between treatment means were compared using Fisher’s Least Significant Difference (LSD) test at a 95% probability level. Statistical analysis was performed using Statgraphics Centurion version 16.1.03.

## 3 Results and discussion

### 3.1 Biomass indicators

The parameters that most reliably define plant N nutritional status are those related to plant growth (Lea and Azevedo, 2006; Iqbal et al., 2020). To evaluate the effect of the biostimulants Pepton 85/16, Pepton Origin, and Nutriterra under different N fertilization levels, we analyzed growth-related parameters, including shoot fresh weight, shoot dry weight, and total leaf area. Shoot biomass (both fresh and dry) reflects overall plant productivity and resource allocation, while leaf area is a key determinant of light interception and photosynthetic capacity, which directly influences plant growth and yield (Marcelis et al., 1998; Poorter et al., 2019). These parameters integrate physiological responses to nutrient availability and biostimulant application and are widely used as reliable indicators to assess plant performance under contrasting nutritional and environmental conditions (Nardi et al., 2016; Rouphael and Colla, 2020).

**Figure 1:**
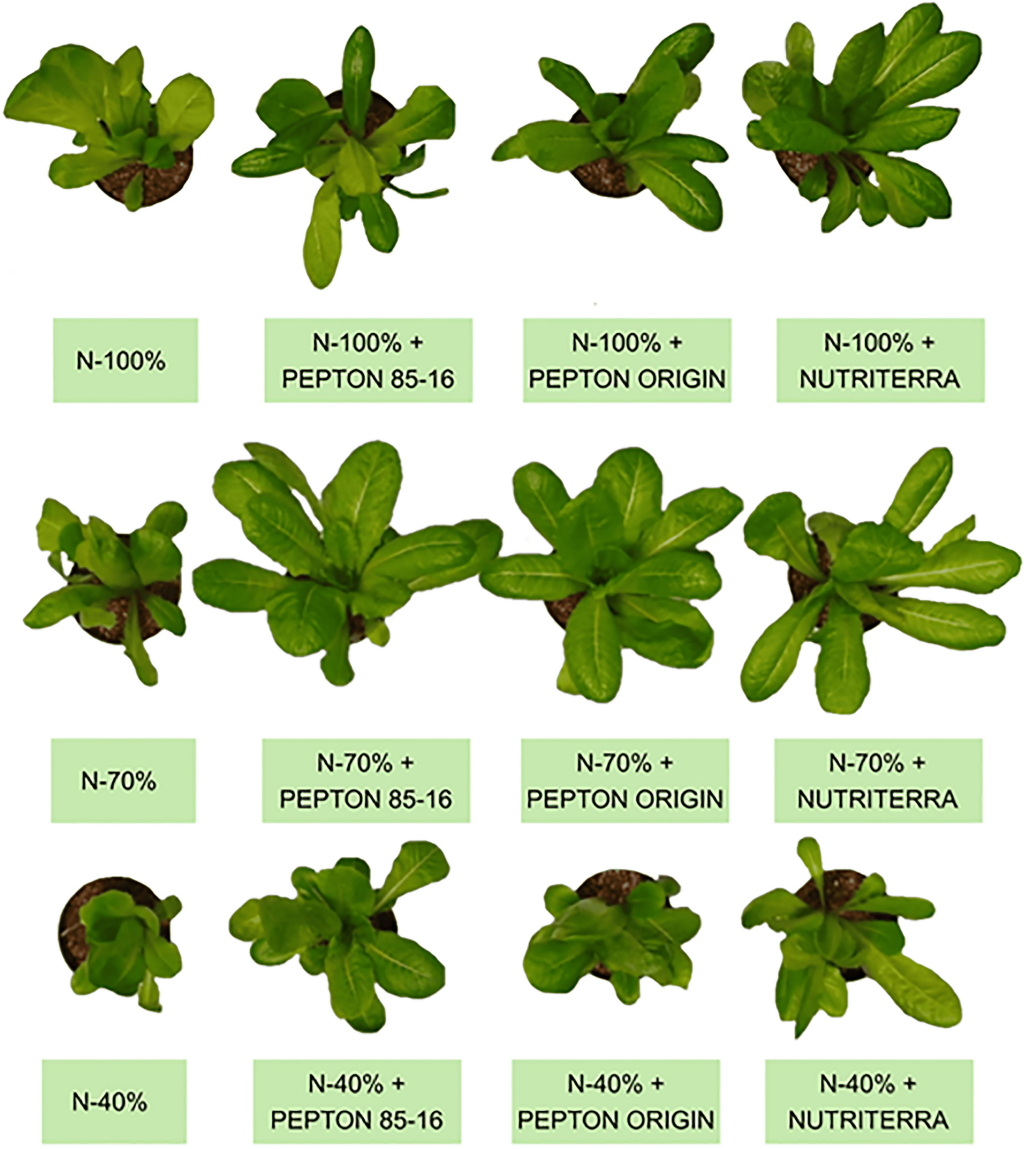
Appearance of lettuce plants subjected to different doses of N fertilization and application of the biostimulants Pepton 85/16, Pepton Origin, and Nutriterra at the time of plant sampling.

Shoot biomass production was reduced under N-limiting conditions (N - 70% and N - 40%) compared to optimal N fertilization (N - 100%), particularly in plants that did not receive any biostimulant. This was expected given the critical role of N in plant growth and development (Iqbal et al., 2020; Lea and Azevedo, 2006) (**Figure 1**; **Table 2**). A general trend was observed across all N treatments when analyzing the effects of biostimulant application: the three biostimulants tested consistently enhanced shoot biomass and leaf area. However, the magnitude of this effect varied with the N level. Under optimal N supply (N - 100%), Nutriterra was the most effective biostimulant, leading to increases of 24%, 23%, and 24% in fresh shoot biomass, dry biomass, and leaf area, respectively, compared to untreated control plants (**Table 2**; **Figure 1**). All biostimulants improved shoot growth and leaf expansion under reduced N conditions (N - 70% and N - 40%), but Pepton 85/16 consistently produced the largest increases. Specifically, under N - 70%, Pepton 85/16 led to 42%, 55%, and 46% increases in fresh shoot biomass, dry biomass, and leaf area, respectively, relative to the control. Under N - 40%, the increases reached 153%, 111%, and 64%, respectively (**Table 2**; **Figure 1**). These increases in biomass are likely attributable to the additional N supplied by the biostimulants, which may compensate for N deficiency, as well as to the presence of other nutrient elements and bioactive compounds (**Table 1**; **Supplementary Table S1**).

**Table 2:** Biomass production parameters and leaf area in lettuce plants subjected to different doses of N fertilization and application of the biostimulants Pepton 85/16, Pepton Origin and Nutriterra at the time of plant sampling.

	Shoot fresh weight (g)	Shoot dry weight (g)	Leaf area (cm^2^)
N-100%	39.49 ± 1.09c	1.57 ± 0.07c	833.02 ± 15.38c
N-100% + PEPTON 85/16	43.48 ± 0.76b	1.66 ± 0.01b	866.92 ± 10.83b
N-100% + PEPTON ORIGIN	42.92 ± 0.74b	1.62 ± 0.03b	879.40 ± 14.28b
N-100% + NUTRITERRA	48.81 ± 0.97a	1.93 ± 0.11a	1036.40 ± 26.46a
*p-value*	<0.001	0.006	<0.001
N-70%	34.07 ± 0.36d	1.39 ± 0.02d	747.95 ± 20.94d
N-70% + PEPTON 85/16	48.37 ± 1.13a	2.16 ± 0.01a	1094.46 ± 49.53a
N-70% + PEPTON ORIGIN	44.52 ± 1.95b	1.81 ± 0.03b	988.73 ± 30.12b
N-70% + NUTRITERRA	39.32 ± 1.11c	1.60 ± 0.04c	910.05 ± 16.25c
*p-value*	<0.001	<0.001	<0.001
N-40%	14.28 ± 0.66d	0.81 ± 0.02d	450.22 ± 8.04d
N-40% + PEPTON 85/16	36.15 ± 0.71a	1.71 ± 0.06a	739.08 ± 11.64a
N-40% + PEPTON ORIGIN	32.50 ± 1.02b	1.41 ± 0.03b	668.12 ± 15.36b
N-40% + NUTRITERRA	27.93 ± 0.33c	1.22 ± 0.02c	643.55 ± 9.25c
*p-value*	<0.001	<0.001	<0.001

Data values represent means ± standard error. Values with different letters indicate significant differences.

Notably, under a 30% N reduction (N - 70%), biostimulant application not only mitigated the negative effects of N deficiency but, in some cases, even surpassed the biomass values recorded under optimal N supply. While Nutriterra restored growth levels comparable to the N - 100% control, both Pepton Origin and, more markedly, Pepton 85/16 induced shoot biomass and leaf area values that exceeded those observed in fully fertilized control plants. These results highlight the potential of biostimulant application, particularly Pepton 85/16, to enhance growth under suboptimal N availability.

The three products used in the study were amino acid-based biostimulants. However, some differences were observed in the plants supplied with each product. The superior performance of Nutriterra under optimal N conditions can be attributed to its high content of humic substances and phosphorus (P) (**Supplementary Table S1**). Humic substances enhance nutrient uptake and stimulate root development by modulating H^+^-ATPase activity and increasing nutrient transporter gene expression, particularly under non-stress conditions (Atero-Calvo et al., 2024a, 2024c). Phosphorus plays a central role in energy metabolism and the regulation of carbon–nitrogen interactions, which may further explain the enhanced growth observed when both N and P are adequately supplied (Marschner, 2012). However, the greater effectiveness of Pepton 85/16 under N-deficient conditions is likely related to its high concentration of free amino acids together with potassium (K) and magnesium (Mg) (**Supplementary Table S1**). Amino acid-rich protein hydrolysates can directly supply organic N and promote N assimilation by stimulating the expression of genes involved in nitrate and ammonium transport and metabolism (Colla et al., 2014; Rouphael and Colla, 2020). Amino acids, such as glutamic acid and proline, may also act as osmoprotectants and signaling molecules under nutrient stress, helping to maintain metabolic activity and cellular homeostasis (Calvo et al., 2014). The presence of K and Mg in Pepton 85/16 may also contribute to improved photosynthetic performance and enzyme activation under conditions of suboptimal N availability (Marschner, 2012). These results are in agreement with those of previous studies on vegetative growth development when Pepton 85/16 was used under different stress conditions (Polo et al., 2006; Marfà et al., 2009; Polo and Mata, 2018; Casadesús et al., 2019, 2020).

### 3.2 Photosynthesis analysis

One of the possible mechanisms of action attributed to the different biostimulants stimulating plant growth is the induction of photosynthesis (Parađiković et al., 2011; du Jardin, 2015). In addition, this physiological process, which is dependent on the synthesis and formation of protein complexes, is highly influenced by fertilization and endogenous N levels in plants (Maathuis, 2009; de Bang et al., 2021; Mu and Chen, 2021). In fact, a higher NUE in plants is usually directly related to an increase in the photosynthetic process by producing a greater assimilation of inorganic N into proteins, such as Rubisco, essential for the photosynthetic CO_2_ fixation process (Ciriello et al., 2024).

Under conditions of adequate fertilization of N (N - 100%), all the tested products increased the *A*, although the application of the Nutriterra product showed the highest values (**Table 3**). These results could explain the increase in biomass produced under 100% N fertilization because of the biostimulant application (**Table 2**). No significant changes were observed in any parameter under optimal conditions of N (N - 100%), with the values of *E*, Ci, and r remaining constant (**Table 3**). Finally, the application of the Nutriterra biostimulant under optimal N conditions was the only one that increased the WUE values (**Table 3**). These data suggest the feasibility of using the Nutriterra product under optimal N conditions, since it significantly improves the use of water to produce a greater amount of biomass in addition to significantly improving the *A* (**Table 3**). The positive results could be attributed to the high content of humic substances and P in Nutriterra. Humic acids stimulate carbon fixation by upregulating key enzymes of the photosynthetic apparatus and enhancing stomatal conductance regulation (Nardi et al., 2002; Atero-Calvo et al., 2024b). Furthermore, P is essential for the production of ATP and NADPH during photosynthesis, and its availability is closely linked to improved CO_2_ assimilation and water productivity (Singh and Raja Reddy, 2011). These synergistic effects likely contribute to a more efficient conversion of resources into biomass under N conditions that are not limited.

**Table 3:** Photosynthetic efficiency at the time of plant sampling in lettuce plants subjected to different doses of N fertilization and application of the biostimulants Pepton 85/16, Pepton Origin and Nutriterra.

	*A* (µmol m^−^² s^−^¹)	*E* (mmol m^−^² s^−^¹)	Ci (µmol mol^−^¹)	r (s cm^-1^)	WUE
N-100%	7.53 ± 0.09b	1.99 ± 0.15	298.54 ± 10.52	5.75 ± 0.26	4.20 ± 0.75
N-100% + PEPTON 85/16	7.41 ± 0.08b	1.92 ± 0.15	303.87 ± 5.75	5.69 ± 0.25	3.97 ± 0.31
N-100% + PEPTON ORIGIN	7.90 ± 0.50b	1.91 ± 0.08	295.28 ± 8.28	5.86 ± 0.06	4.15 ± 0.29
N-100% + NUTRITERRA	10.93 ± 0.14a	2.11 ± 0.07	304.81 ± 3.95	5.72 ± 0.15	5.21 ± 0.14
*p-value*	<0.001	0.145	0.111	0.292	0.174
N-70%	6.32 ± 0.18d	2.34 ± 0.08	308.07 ± 4.31b	5.60 ± 0.13	2.71 ± 0.10c
N-70% + PEPTON 85/16	10.20 ± 0.22a	2.27 ± 0.08	313.92 ± 1.97ab	5.38 ± 0.30	4.51 ± 0.12a
N-70% + PEPTON ORIGIN	9.25 ± 0.11b	2.20 ± 0.13	316.37 ± 4.70ab	5.62 ± 0.20	4.27 ± 0.23a
N-70% + NUTRITERRA	7.49 ± 0.27c	2.30 ± 0.07	323.25 ± 6.05a	5.52 ± 0.16	3.29 ± 0.24b
*p-value*	<0.001	0.285	0.035	0.271	<0.001
N-40%	3.46 ± 0.31c	0.90 ± 0.03c	120.44 ± 9.48c	10.73 ± 0.29a	3.91 ± 0.46c
N-40% + PEPTON 85/16	8.33 ± 0.16a	1.39 ± 0.14ab	220.31 ± 2.41a	7.80 ± 1.06b	6.34 ± 0.64a
N-40% + PEPTON ORIGIN	6.81 ± 0.13b	1.57 ± 0.08a	214.93 ± 1.97a	7.91 ± 0.91b	4.39 ± 0.21c
N-40% + NUTRITERRA	6.25 ± 0.38b	1.16 ± 0.04b	195.05 ± 6.75b	8.32 ± 0.71b	5.44 ± 0.42b
*p-value*	<0.001	<0.001	<0.001	0.0197	0.009

Net photosynthetic rate (*A*), transpiration rate (*E*), intracellular CO_2_ concentration (Ci), stomatal resistance (r), and water use efficiency (WUE). Data values represent means ± standard error. Values with different letters indicate significant differences.

When the application of N was limited in the culture medium (N - 70% and N - 40%), all the biostimulants applied improved the *A* in plants (**Table 3**). However, under these conditions of N limitation, the Pepton Origin biostimulants and especially Pepton 85/16 stand out especially for the *A* induction they produce, with the maximum values being presented with the Pepton 85/16 treatment (**Table 3**), which correlates with the increase in plant growth (**Table 2**).

Under N-limiting conditions, especially in severe deficiencies, gas exchange in plants is usually affected by higher stomatal closure because the lack of N in the culture medium affects water absorption by reducing root extension and the synthesis of specific water transporters such as aquaporins (Maathuis, 2009; de Bang et al., 2021; Mu and Chen, 2021). In this study, gas exchange was only affected when the reduction in N application in the culture medium was 60% (N - 40%) (**Table 3**). In this situation, in the control plants, there was a greater stomatal closure (higher values of r), and therefore, *E* and Ci were significantly reduced (**Table 3**). On the contrary, under these conditions, the application of the biostimulants Pepton 85/16, Pepton Origin, and to a lesser extent Nutriterra reduced in stomatal closure with lower r values, which generated a higher rate of *E* and Ci, which could explain the higher rates of *A* obtained with the application of these products and especially with the application of Pepton 85/16 (**Table 3**). Finally, when the reduction in the application of N to the medium was 30% (N - 70%), all treatments presented similar values of gas exchange (*E*, Ci, and r), with no statistical differences compared with the control plants (**Table 3**).

Under N-deficient conditions, the increased *A* observed with Pepton 85/16 and Pepton Origin may be linked to the presence of specific amino acids, such as glutamic acid, proline, and glycine, which are known to enhance N assimilation and stimulate Rubisco activity even under nutrient stress (Batista-Silva et al., 2019). These amino acids can act as signaling molecules and metabolic intermediates that sustain photosynthetic performance when N supply is limited. Moreover, the observed increase in *E* and Ci suggests that these protein hydrolysate-based biostimulants promote stomatal conductance and carbon uptake efficiency, a mechanism previously reported in crops treated with amino acid-rich formulations under stress conditions (Colla et al., 2015). This may explain the enhanced *A*, particularly in plants treated with Pepton 85/16, despite the suboptimal N environment.

Regarding the WUE values under N-limiting conditions in the culture medium (N - 70% and N - 30), we observed that the application of all biostimulants significantly improved the values of this parameter compared to those obtained in control plants (**Table 3**), the most beneficial being the Pepton 85/16 product (**Table 3**). These data indicate that the use of the biostimulants Pepton 85/16, Pepton Origin, and Nutriterra under N-deficient conditions is an effective strategy to improve water use in biomass production, with Pepton 85/16 being the most effective product under these conditions. Similar results were obtained when Pepton 85/16 was applied to tomatoes growing under water restrictions (Casadesús et al., 2019).

The photosynthesis results would explain the biostimulant effect produced in lettuce plants by the application of the products Pepton 85/16, Pepton Origin and Nutriterra, defining the products Nutriterra, under adequate conditions of N, and Pepton 85/16, under deficient conditions of N, as the most effective in terms of their potentiating effect of the photosynthetic process. These results would explain why the application of these biostimulants (especially Nutriterra and Pepton 85/16) under different conditions of N availability in the culture medium significantly improves plant growth and biomass production (**Table 2**), which would define this physiological process as one of the main mechanisms of action of these products.

### 3.3 N metabolism and enzymatic activities

Some research has shown that the application of biostimulants based on the presence of amino acids can improve plant growth and productivity by stimulating the assimilation efficiency of certain essential nutrients such as N (Di Mola et al., 2020). Recent research indicates that the use of biostimulants based on the application of amino acids leads to an increase in the assimilation of N through the induction of enzymes such as NR and GS, both of which are the main regulators of this physiological process (Colla et al., 2017; Ottaiano et al., 2021).

**Figure 2:**
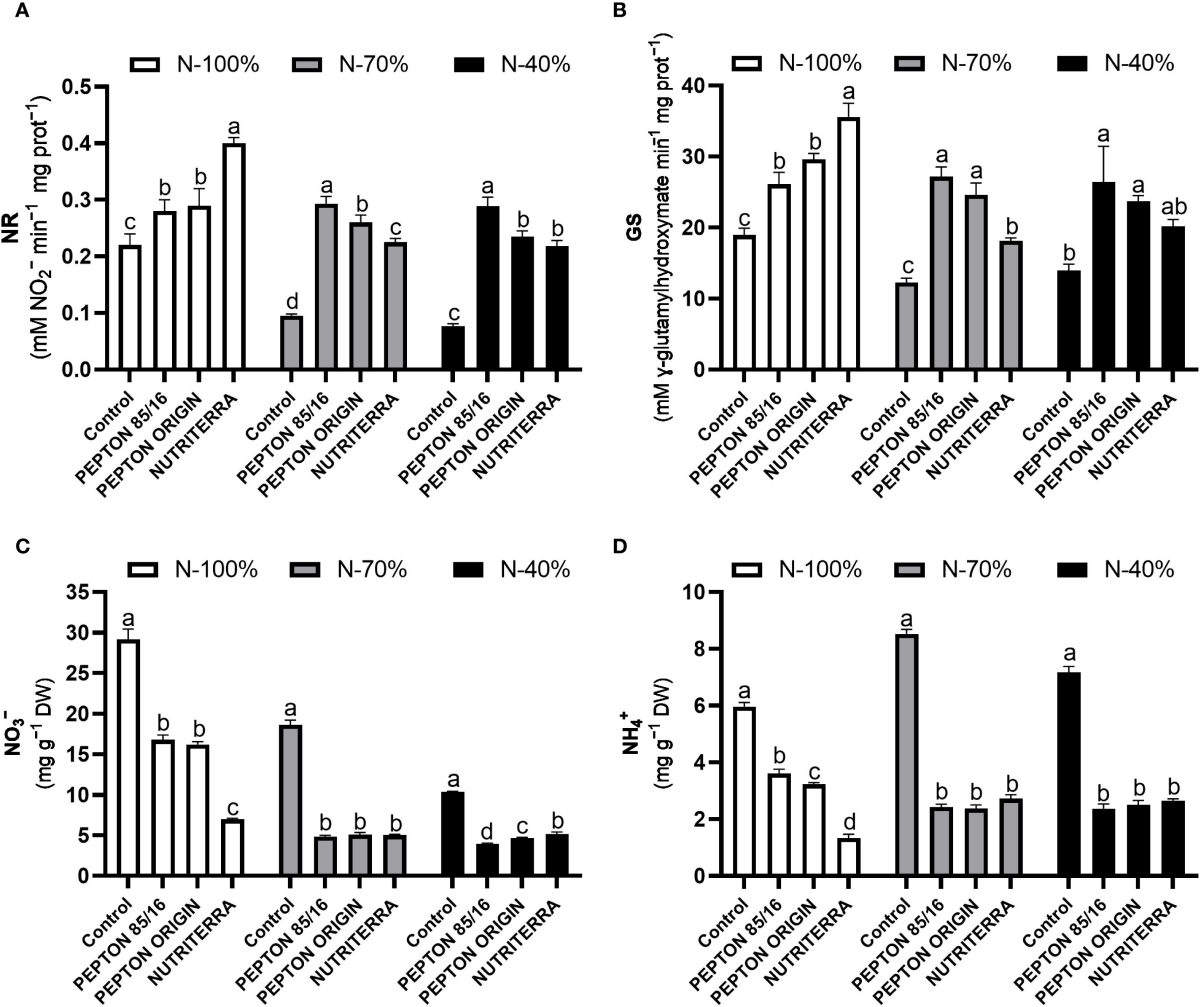
Nitrate reductase (NR) activity **(A)**, glutamine synthetase (GS) activity **(B)**, NO_3_^-^ concentration **(C)**, and NH_4_^+^ concentration **(D)** in lettuce plants subjected to different N fertilization levels and biostimulant treatments. Columns represent the means and error bars, standard error. Values with different letters indicate significant differences.

In this study, the use of all the tested biostimulants produced an induction of NR and GS enzyme activities, regardless of the dose of N applied in fertilization. These enzymatic activities were always higher than those of the control plants. Under N - 100% fertilization, the biostimulant that produced the maximum values of NR and GS was Nutriterra, while under limiting conditions in fertilization with N (N - 70% and N - 40%), the application of Pepton 85/16 gave rise to the maximum NR and GS activities in the plants (**Figures 2A, B**).

Knowing the concentration status of the inorganic forms of N, such as NO_3_^-^ and NH_4_^+^, is essential for explaining the processes of N assimilation. In general, the reduction in the concentration of NO_3_^-^ and NH_4_^+^ in the leaves is usually indicative of a stimulation of the processes of N assimilation, since this decrease is directly related to the induction of the main enzymes such as NR and GS, that carry out this physiological process (Maathuis, 2009; Vidal et al., 2020). Indeed, the application of the different biostimulants under N - 100%, N - 70%, and N - 40%, led to a very significant reduction in the foliar concentration of NO_3_^-^ and NH_4_^+^, especially in the extreme conditions of limited application of N in the medium, since Nutriterra and Pepton 85/16, respectively, had a more significant effect on the reduction in leaf concentration of these inorganic forms of N in N - 100% and N - 40% (**Figures 2C, D**). The reduction in NO_3_^-^ and NH_4_^+^ concentrations observed with the application of biostimulants correlates with the increase in NR and GS activities in these treatments (**Figures 2A, B**).

In short, these results indicate that both biostimulants, Nutriterra under conditions of N - 100% and Pepton 85/16 under limiting conditions of N (N - 70% and N - 40%) in the culture medium, enhance both the process of NO_3_^-^ reduction and the incorporation of NH_4_^+^ into amino acids, which could be defined as a mechanism of action that would explain the higher rates of photosynthesis presented in these treatments, a process that is highly dependent on the generation of organic N compounds (Maathuis, 2009; de Bang et al., 2021; Mu and Chen, 2021). In addition, it would explain the induction of greater plant growth since N assimilation is an essential process closely linked to plant biomass production (de Bang et al., 2021).

Finally, leaf NO_3_^−^ content indicates the nutritional quality of lettuce, particularly when N is predominantly supplied as NO_3_^−^, as in this study. Monitoring foliar NO_3_^−^ is critical in leafy vegetables, since excessive accumulation poses health risks due to its conversion into nitrite and N-nitroso compounds, which have been associated with methemoglobinemia and increased cancer risk through nitrosamine formation (Mensinga et al., 2003). Our results demonstrate that the application of the three biostimulants effectively reduced leaf NO_3_^−^ accumulation in lettuce (**Figure 2C**). This outcome adds value to lettuce production by improving its nutritional quality and reducing the potential health risks associated with high NO_3_^−^ intake. Notably, Nutriterra was the most effective at lowering foliar NO_3_^−^ content under optimal N supply (N - 100%) (**Figure 2C**). Biostimulants may enhance NO_3_^−^ assimilation or redistribution by stimulating enzymatic activity involved in N metabolism, such as NR, as reported by du Jardin (2015) and Rouphael and Colla (2020). Consequently, the use of these products may contribute to safer and higher-quality leafy vegetables without compromising yield.

### 3.4 N accumulation and N assimilation products

The incorporation of NH_4_^+^ can be quantified by analyzing soluble proteins and amino acids. Therefore, these parameters are crucial for determining the nitrogenous nutritional status of plants (Iqbal et al., 2020; The et al., 2021). Regarding the concentration of soluble proteins, it was again the application of all biostimulants that determined the maximum concentrations, both under suitable conditions (N - 100%) and limiting fertilization of N (N - 70% and N - 40%) (**Table 4**). This increment is directly proportional to the increase produced by the application of these products in the enzymatic activities responsible for the assimilation of N (**Figure 2**). The increase in soluble proteins in the different doses of N used in fertilization, especially with the application of Nutriterra under a fertilization of N - 100% and Pepton 85/16 in limiting conditions of N (N - 70% and N - 40%), both with the maximum values, would also explain the increase in plant growth that the application of these biostimulants entails (**Table 2**) since soluble proteins represent the levels of cellular enzymes involved in essential processes such as N assimilation, photosynthesis, carbon metabolism, etc (Iqbal et al., 2020; The et al., 2021).

**Table 4:** Concentration of N forms at the time of plant sampling in lettuce plants subjected to different doses of N fertilization and application of the biostimulants Pepton 85/16, Pepton Origin and Nutriterra.

	Proteins (mg g^-1^ FW)	Amino acids (mg g^-1^ FW)	Organic N (mg g^-1^ DW)	Total N (mg g^-1^ DW)
N-100%	2.40 ± 0.10c	0.51 ± 0.02c	64.67 ± 1.56c	93.85 ± 2.51
N-100% + PEPTON 85/16	3.93 ± 0.22b	0.73 ± 0.02b	75.07 ± 1.13b	91.83 ± 1.41
N-100% + PEPTON ORIGIN	3.62 ± 0.28b	0.72 ± 0.02b	74.73 ± 1.06b	90.93 ± 1.35
N-100% + NUTRITERRA	6.16 ± 0.27a	0.81 ± 0.03a	86.87 ± 2.02a	93.84 ± 2.04
*p-value*	<0.001	<0.001	<0.001	0.195
N-70%	1.51 ± 0.11d	0.38 ± 0.02d	53.56 ± 1.78d	72.17 ± 1.82c
N-70% + PEPTON 85/16	5.35 ± 0.17a	0.79 ± 0.01a	83.07 ± 1.65a	87.90 ± 1.62a
N-70% + PEPTON ORIGIN	4.67 ± 0.12b	0.73 ± 0.01b	76.14 ± 1.72b	81.22 ± 1.80b
N-70% + NUTRITERRA	4.05 ± 0.18c	0.56 ± 0.02c	67.05 ± 1.06c	72.07 ± 1.02c
*p-value*	<0.001	<0.001	<0.001	<0.001
N-40%	1.21 ± 0.14d	0.21 ± 0.01d	44.43 ± 0.94c	54.79 ± 0.97c
N-40% + PEPTON 85/16	4.10 ± 0.12a	0.55 ± 0.04a	69.09 ± 1.50a	73.02 ± 1.43a
N-40% + PEPTON ORIGIN	3.62 ± 0.16b	0.47 ± 0.03b	64.73 ± 2.08a	69.40 ± 2.05a
N-40% + NUTRITERRA	3.05 ± 0.12c	0.39 ± 0.01c	53.24 ± 3.20b	58.41 ± 3.16b
*p-value*	<0.001	<0.001	<0.001	<0.001

Data values represent means ± standard error. Values with different letters indicate significant differences.

The analysis of soluble amino acids is essential not only because of their importance in plant growth but also because of their regulatory effect on the N absorption and assimilation processes (Iqbal et al., 2020; The et al., 2021). The application of all the biostimulants tested produced a significantly greater accumulation of soluble amino acids compared to control plants (**Table 4**). More specifically, and differentiating the doses of N applied, Nutriterra with a fertilization of N - 100% and Pepton 85/16 with limiting conditions of N (N - 70% and N - 40%) were the treatments that determined the maximum concentration of soluble amino acids in the plants (**Table 4**), which indicates a significant stimulation of the N assimilation process.

Finally, all biostimulants increased organic N concentrations under full N supply (N - 100%), with Nutriterra being the most effective. This effect, supported by the increases in soluble proteins, amino acids (**Table 4**), and NR and GS (**Figures 2A, B**), suggests that N assimilation is significantly promoted by the application of these biostimulants. This is in line with previous studies showing that biostimulants based on protein hydrolysates or humic substances enhance the activity of key N-assimilation enzymes and stimulate amino acid biosynthesis under adequate nutrition (Colla et al., 2015; Ciriello et al., 2024).

Under N-limited conditions (N - 70% and N - 40%), biostimulant application maintained or increased organic N levels relative to the control. Pepton 85/16 induced the highest concentrations of organic N, proteins, and amino acids (**Table 4**), as well as greater NR and GS activity (**Figures 2A, B**), indicating a strong stimulation of N assimilation even under suboptimal supply. Notably, the total N content was significantly increased by Pepton Origin and, more markedly, Pepton 85/16 under N-limited conditions, suggesting improved N uptake and accumulation efficiency. These results could be due to the higher N content of these two biostimulants than that of Nutriterra (**Table 1** and **Supplementary Table S1**). In addition, these results may be explained by the presence of signaling peptides and bioactive amino acids in protein hydrolysates, which have been shown to enhance root development, increase transporter activity, and modulate N metabolism genes (Lucini et al., 2015). In previous studies under abiotic stress conditions Pepton 85/16 was able to modify the plant hormones expression to improve vegetative growth and increase root development (Casadesús et al., 2019, 2020).

From a physiological perspective, the superior performance of Pepton 85/16 under N-limiting conditions may be attributed to its high glutamic acid and aspartic acid content, which are two amino acids closely linked to N assimilation and transamination reactions (Alfosea-Simón et al., 2021). In addition, potassium oxide content may enhance N uptake by improving ionic balance and stomatal function under stress (Wang et al., 2013). The high content of phenylalanine and other aromatic amino acids (tryptophan and tyrosine) in Pepton 86/16 has been suggested as a precursor of salicylic acid, which is involved in defense response against biotrophic pathogens. However, low concentrations can promote lateral root development that can help in N assimilation (Casadesús et al., 2020).

### 3.5 NUE parameters

Considering all the problems associated with N fertilizers, agronomic techniques that lead to an improvement in NUE by plants are essential (Xu et al., 2012). Therefore, higher NUE could enhance crop yield and quality, reduce economic costs, and decrease environmental degradation caused by the application of N fertilizers. One of the strategies currently being analyzed to improve the NUE of agricultural crops is the application of biostimulants, specifically those based on amino acid mixtures. Thus, Navarro-León et al. (2022) observed that the application of biostimulants composed of amino acids increased the concentration of total N and NUE indices such as NUpE and NUtE in lettuce plants subjected to different doses of N (100%, 60% and 30% N). Similar results have also been found in basil plants (*Ocimun basilicum* L.), in which the application of this type of biostimulant increased NUE by more than 15% both in optimal and deficient conditions of N (0 and 50 Kg N/Ha) (Consentino et al., 2023).

**Figure 3:**
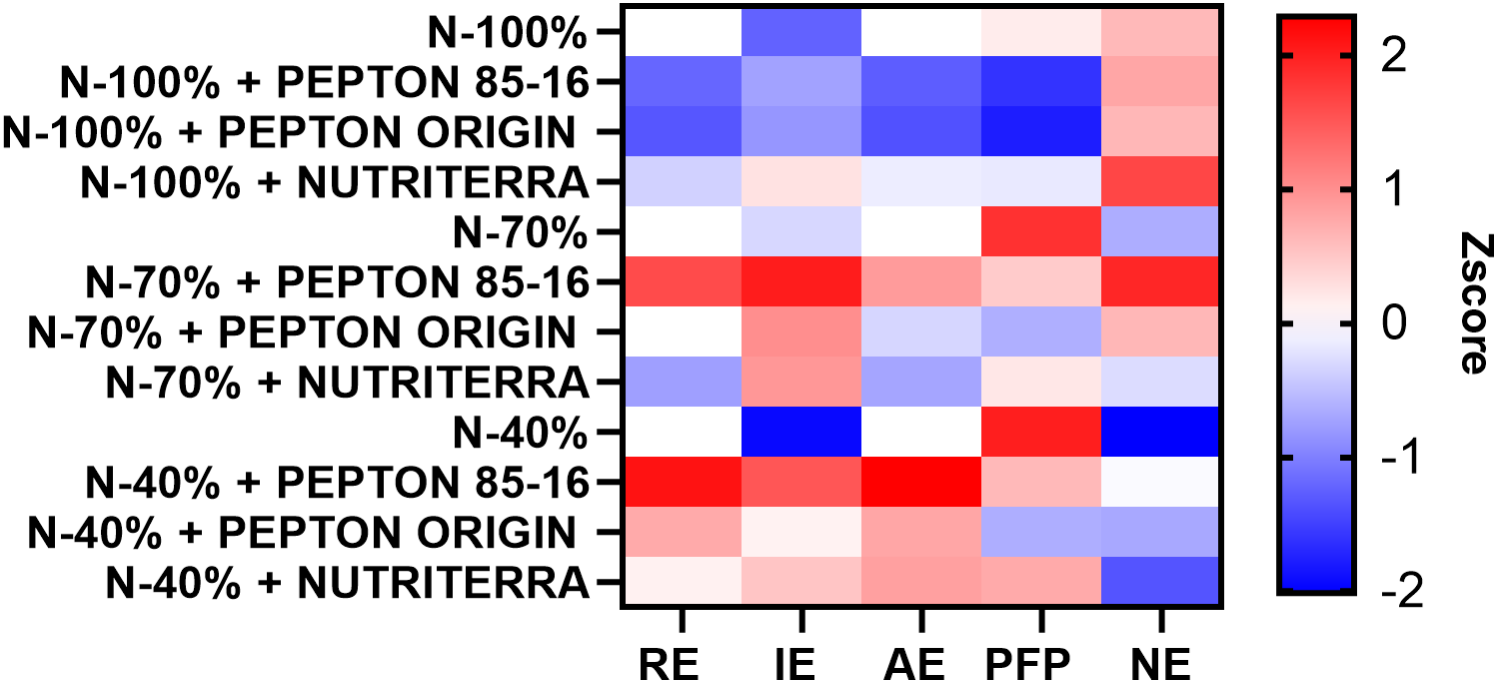
Heatmap showing the Z-score normalized values of five N use efficiency parameters in lettuce plants subjected to different N fertilization levels and biostimulant treatments. Parameters include apparent N recovery efficiency (RE), internal N utilization efficiency (IE), agronomic efficiency of applied nitrogen (AE), partial factor productivity of applied nitrogen (PFP), and nutrient export (NE). Red and blue colors indicate higher and lower values, respectively, based on Z-score scaling.

Under optimal N fertilization (N - 100%), the application of the biostimulant Nutriterra resulted in the most favorable values for all NUE indices (**Figure 3**; **Supplementary Table S1**). Notably, Nutriterra significantly increased NE, indicating a better alignment between N uptake and aboveground N accumulation. Moreover, this treatment resulted in the highest values of IE and AE, reflecting an enhanced capacity of the crop to translate N inputs into biomass. These findings suggest that Nutriterra can substantially improve N utilization under intensive cropping systems. Previous studies have reported similar effects on NUE through enhanced photosynthetic performance, protein synthesis, and N assimilation in crops treated with amino acid- and metabolite-rich biostimulants (Colla et al., 2015). By improving NUE, Nutriterra contributes not only to greater productivity but also to more sustainable fertilization strategies, potentially reducing the need for excessive N application and limiting associated environmental impacts such as nitrate leaching or greenhouse gas emissions (Govindasamy et al., 2023; Ali et al., 2025).

Under N-limited conditions (N - 70% and N - 40%), the most effective improvements in NUE indices were obtained with the application of Pepton 85/16, followed by Pepton Origin (**Figure 3**). Pepton 85/16 consistently increased NE, IE, and AE, and showed the most favorable RE values, demonstrating a high capacity to enhance both N uptake and physiological use under suboptimal fertilization. This response is likely related to its high content of bioactive peptides and N-rich amino acids, such as glutamic and aspartic acid, which are closely involved in N transport and assimilation processes (Calvo et al., 2014). In a study conducted applying Pepton 85/16 combined with low-N priming in the production and quality of greenhouse tomatoes, Pepton 85/16 was found to increase the yield of greenhouse NUE tomatoes, showing an additive effect with low-N priming, without negatively affecting fruit quality (Mesa et al., 2022).

The improvement of NUE under N-deficient conditions is of particular relevance from an agroecological perspective. Strategies that increase NUE can significantly reduce the environmental footprint of cropping systems by minimizing fertilizer input while maintaining or even increasing yield (Ciampitti and Vyn, 2012; Zhang et al., 2015). In this sense, Pepton 85/16 may represent a valuable tool for improving the sustainability of lettuce production, especially in systems that aim to reduce the use of N fertilizer without compromising crop performance.

## 4 Conclusions

Overall, the results of this study indicate that the application of the three biostimulants from APC Agro (Pepton 85/16, Pepton Origin, and Nutriterra) significantly enhanced aboveground plant growth under both optimal N fertilization (N - 100%) and N-limited conditions (N - 70% and N - 40%). This positive effect, in addition to the N supplied by the biostimulants, appears to be associated with these physiological mechanisms: stimulation of photosynthesis, enhanced N assimilation through higher NR and GS enzymatic activities, elevated protein and amino acid concentrations, and improved NUE, both in terms of N uptake and physiological utilization. Nutriterra was the most effective under full N supply, making it suitable for use in intensive cropping systems with no N limitations. Pepton 85/16 was the most effective biostimulant under N-deficient conditions. Its application not only improved N uptake and use but also led to greater plant growth, particularly under a 30% N reduction (N - 70%), where it fully restored and even enhanced biomass production compared to the optimal N condition. Although Pepton Origin also showed positive effects under N-limited conditions, its efficacy was slightly lower than that of Pepton 85/16. Overall, the use of Pepton 85/16 could allow for a reduction in N fertilizer inputs in systems where N is not limiting, without compromising crop performance, thus offering a valuable strategy to reduce costs and mitigate the environmental impact of intensive fertilization.

## Data Availability

The raw data supporting the conclusions of this article will be made available by the authors, without undue reservation.
