# Supplementary material for: Physiological efficacy of the amino acid-based biostimulants Pepton 85/16, Pepton origin, and Nutriterra in lettuce grown under optimal and reduced synthetic nitrogen fertilization
Source: Front Plant Sci. 2025 Sep 8;16:1645768. doi: 10.3389/fpls.2025.1645768 (PMC12450945; doi:10.3389/fpls.2025.1645768)
Supplement: Supplementary file 1 [file DataSheet1.docx]

| **Component** | **NutriTerra** | **Pepton Origin** | **Pepton 85/16** |
| --- | --- | --- | --- |
| Total amino acids | 13.50 | 93.00 | 85.00 |
| Free amino acids | <2.00 | ≥ 40.00 | ≥16.00 |
| Alanine | 1.16 | 7.50 | 7.17 |
| Methionine | 0.14 | 0.80 | 0.70 |
| Arginine | 0.20 | 3.00 | 3.33 |
| Phenylalanine | 1.02 | 6.10 | 5.73 |
| Aspartic acid | 1.87 | 11.00 | 10.74 |
| Serine | 0.24 | 4.10 | 3.25 |
| Cysteine | 0.08 | 0.50 | <0.10 |
| Threonine | 0.06 | 2.90 | 2.66 |
| Glutamic acid | 1.42 | 7.30 | 7.35 |
| Tryptophan | 0.16 | 1.40 | 1.21 |
| Glycine | 0.82 | 4.30 | 4.43 |
| Tyrosine | 0.44 | 2.00 | 1.64 |
| Histidine | 0.95 | 7.40 | 5.78 |
| Valine | 1.39 | 8.30 | 7.90 |
| Isoleucine | 0.18 | 0.40 | 0.35 |
| Proline | 0.58 | 3.10 | 3.09 |
| Leucine | 1.92 | 12.00 | 12.16 |
| Hydroxyproline | <0.04 | <0.04 | <0.04 |
| Lysine | 1.04 | 7.70 | 7.26 |
| Total nitrogen (N) | 2.20 | 14.00 | 13.00 |
| Phosphorus pentoxide (P₂O₅) | 3.00 | 0.70 | 0.70 |
| Potassium oxide (K₂O) | 3.60 | 1.49 | 5.00 |
| Humic extract | 14.00 |  |  |
| Humic acids | 1.00 |  |  |
| Fulvic acids | 13.00 |  |  |
| Calcium oxide (CaO) | 0.02 | 0.04 | 0.03 |
| Iron (Fe) | 0.03 | 0.27 | 0.20 |
| Magnesium oxide (MgO) |  |  | 0.04 |

**Table S1**. Composition of the three biostimulants used in the study. The values of Pepton Origin and Pepton 85/16 expressed as percentage weight per weight (% w/w) and the values of NutriTerra are expressed as percentage weight per volume (% w/v)

Table S2. Nitrogen use efficiency parameters at the time of plant sampling in lettuce plants subjected to different nitrogen fertilization rates and the application of the biostimulants Pepton 85-16, Pepton Origin, and Nutriterra.

|  | **RE** (mg mg^−1^) | **IE** (g² DW mg^−1^ N) | **AE** (mg DW mg^−1^ N) | **PFP** (mg DW mg^−1^ N) | **NE** (mg N) |
| --- | --- | --- | --- | --- | --- |
| **N-100%** |  | 0.0167 ± 0.0009c |  | 3.73 ± 0.17a | 146.89 ± 7.64b |
| **N-100% + PEPTON 85/16** | 0.008 ± 0.014b | 0.0181 ± 0.0003b | 0.14 ± 0.12b | 2.44 ± 0.02b | 152.57 ± 2.45b |
| **N-100% + PEPTON ORIGIN** | 0.001 ± 0.011b | 0.0179 ± 0.0005b | 0.08 ± 0.12b | 2.32 ± 0.04b | 147.44 ± 3.30b |
| **N-100% + NUTRITERRA** | 0.061 ± 0.022a | 0.0207 ± 0.0015a | 0.86 ± 0.15a | 3.50 ± 0.20a | 180.42 ± 8.34a |
| ***p-value*** | 0.04 | 0.008 | 0.001 | <0.001 | 0.002 |
|  |  |  |  |  |  |
| **N-70%** |  | 0.0193 ± 0.0007c |  | 4.71 ± 0.08a | 99.84 ± 2.13d |
| **N-70% + PEPTON 85/16** | 0.163 ± 0.007a | 0.0246 ± 0.0005a | 1.40 ± 0.04a | 3.90 ± 0.03b | 189.96 ± 3.20a |
| **N-70% + PEPTON ORIGIN** | 0.083 ± 0.009b | 0.0224 ± 0.0005b | 0.75 ± 0.04b | 3.16 ± 0.05c | 147.36 ± 4.64b |
| **N-70% + NUTRITERRA** | 0.036 ± 0.010c | 0.0222 ± 0.0006b | 0.50 ± 0.10c | 3.75 ± 0.09b | 115.18 ± 3.12c |
| ***p-value*** | <0.001 | <0.001 | <0.001 | <0.001 | <0.001 |
|  |  |  |  |  |  |
| **N-40%** |  | 0.0148 ± 0.0004c |  | 4.81 ± 0.12a | 44.29 ± 1.39d |
| **N-40% + PEPTON 85/16** | 0.188 ± 0.009a | 0.0235 ± 0.0010a | 2.11 ± 0.13a | 4.00 ± 0.14b | 124.78 ± 4.44a |
| **N-40% + PEPTON ORIGIN** | 0.121 ± 0.010b | 0.0204 ± 0.0004b | 1.35 ± 0.05b | 3.15 ± 0.06c | 98.31 ± 4.58b |
| **N-40% + NUTRITERRA** | 0.089 ± 0.007c | 0.0213 ± 0.0014ab | 1.38 ± 0.12b | 4.07 ± 0.07b | 71.10 ± 3.01c |
| ***p-value*** | <0.001 | <0.001 | <0.001 | <0.001 | <0.001 |

Apparent N recovery efficiency (RE), internal N utilization efficiency (IE), agronomic efficiency of applied N (AE), partial factor productivity of applied N (PFP), and nutrient export (NE). Data values represent means ± standard error. Values with different letters indicate significant differences.
